# MAPK15 controls mitochondrial fitness and contributes to prevent cellular senescence

**DOI:** 10.1080/27694127.2022.2113016

**Published:** 2022-08-23

**Authors:** Lorenzo Franci, Giovanni Inzalaco, Mario Chiariello

**Affiliations:** a Istituto di Fisiologia Clinica (IFC), Consiglio Nazionale delle Ricerche (CNR), and Core Research Laboratory (CRL), Istituto per lo Studio, la Prevenzione e la Rete Oncologica (ISPRO), Siena, Italy; b Department of Medical Biotechnologies, University of Siena, Siena, Italy

**Keywords:** cell homeostasis, MAPK, mitophagy, oxidative stress, senescence

## Abstract

Aberrant production of reactive oxygen species (ROS) from dysfunctional mitochondria leads to oxidative stress and DNA damage, which induces the cellular senescence stress response pathway. This, while exerting strong beneficial suppressive effects on the development of cancer, also contributes to aging and different age-related disorders. Mitophagy is a key mechanism to constantly eliminate old and damaged mitochondria, strongly contributing to keep low levels of intracellular ROS. Here, we discuss our recent findings showing the involvement of the atypical MAP kinase family member MAPK15 in controlling the mitophagic process, thereby preventing ROS accumulation, extensive DNA damage and activation of the cellular senescence phenotype.

Persistent DNA damage is considered a key mechanism for the establishment of the cellular senescent phenotype, a physiological state whereby cells fail to reenter the cell cycle under mitotic stimuli and manifest an enhanced secretory phenotype, ultimately contributing to organismal aging. ROS are an important cause of DNA damage and are endogenously produced by mitochondria, peroxisomes and endoplasmic reticulum, but external stimuli such as ionizing radiation, chemotherapeutic drugs and environmental insults may also contribute to their accumulation. Particularly, mitochondria “physiologically” generate small amounts of ROS as a byproduct of oxidative phosphorylation (OXPHOS). Still, old or exogenously damaged mitochondria may become much less efficient and increase ROS production to an extent that can became toxic to the cell. For this reason, cells have developed an efficient mechanism to remove damaged mitochondria through mitophagy, which exerts a constant quality control over these organelles. As oxidative damage due to altered mitophagy and mitochondrial dysfunctions have been implicated in human age-related diseases, understanding of the complex mechanisms used by cells to control these processes is surely of great interest and promise the development of new therapeutic approaches to improve human health.

With the aim of studying the ability of MAPK15/ERK7/ERK8 (mitogen-activated protein kinase 15) to regulate cellular metabolism, in Franci et al. [1] we first observed that downregulating the expression of this gene strongly decreases ATP production, mainly affecting OXPHOS. As enhanced levels of mitochondrial ROS (mt-ROS) are a frequent consequence of an inefficient mitochondrial respiration, we next evaluated the correlation of mt‐ROS levels with MAPK15 protein expression, both in unstimulated conditions and upon exposure to two different mitochondria-damaging toxins, rotenone and carbonyl cyanide 4‐(trifluoromethoxy) phenylhydrazone (FCCP). Indeed, in all these conditions, MAPK15 expression is inversely correlated with mt-ROS levels, upon both overexpression and siRNA-mediated downregulation of the gene, overall implying that MAPK15 is necessary to counteract superoxide production from mitochondria. Interestingly, a MAPK15 mutant, which is specifically deficient in its autophagic function, is not able to reduce mt-ROS in unstimulated or rotenone or FCCP‐treated cells, indicating that MAPK15 autophagic function is critical to counteract ROS production. Consequently, we hypothesized that MAPK15 may have a role in regulating the elimination of damaged mitochondria through the selective autophagic mechanism of mitophagy, maintaining the fitness of the mitochondrial “compartment”. Indeed, MAPK15-downregulated cells show increased mitochondrial mass (because of defective elimination) and reduced accumulation of clusters of mitochondria in the peri-nuclear area, which often precedes their mitophagic elimination. Consistently, MAPK15 overexpression strongly reduces the mass and increases the peri-nuclear aggregation of mitochondria, effects that are prevented by overexpressing autophagy- and kinase-deficient mutants of the gene. Supporting a positive role of MAPK15 in the control of mitophagic flux, we also demonstrated that, in basal and FCCP-treated conditions, the overexpressed kinase increases mitochondria colocalization with autophagosomes and autolysosomes. In line with these results, MAPK15 downregulation also reduces mitophagic flux when assessed by a mitochondrial matrix-targeted fluorescent Keima reporter protein (mt-Keima) assay. Interestingly, at the molecular level, we demonstrated that MAPK15 takes advantage of the AMPK-ULK1 signaling pathway to induce PRKN activating phosphorylation, an event already demonstrated to induce mitophagy.

Increased amounts of mt-ROS, detected in MAPK15 downregulated cells as a consequence of impaired mitophagy, may therefore contribute to extensive DNA damage and, consequently, induce protective cell responses aimed at preventing transformation, including cellular senescence. Indeed, in different model tumor cells, we observe strong induction of DNA damage, cell cycle arrest and cellular senescence. Importantly, these effects can be all partially rescued by mito-TEMPO, a specific mt-ROS scavenger, overall suggesting that mt-ROS produced as a consequence of MAPK15 downregulation and accumulation of damaged mitochondria are clearly contributing to these events.

As cellular senescence is a key mechanism involved in preventing normal cells from undergoing transformation, we ultimately looked for confirmation of our results in primary normal human airway epithelial cells which, in vivo, are constantly subjected to external insults such as cigarette smoke and environmental pollution. Indeed, downregulation of MAPK15 expression in these cells showed the presence of all markers of cellular senescence: reduced cell proliferation with increased CDKN1A/p21 protein levels, increased senescence-associated GLB1/β-galactosidase activity, extensive nuclear DNA damage scored by counting the number of γ-H2AX foci and telomere-associated DNA damage foci (TAF), and increased production of different cytokines accompanying the senescence-associated secretory phenotype, i.e. CCL2, CXCL1, CXCL2, IL6 and IL8.

Altogether, our data support a key role for MAPK15 in controlling the homeostasis of the mitochondrial compartment. Importantly, the function of this protein is important both to counteract “physiological” ageing of these organelles, and to eliminate mitochondria acutely damaged by extracellular toxins. Reduced expression or activity of the kinase, therefore, produce an accumulation of old and damaged mitochondria, which became the source of mutagenic ROS representing the trigger for the induction of cellular senescence (Figure 1).

**Figure 1.**
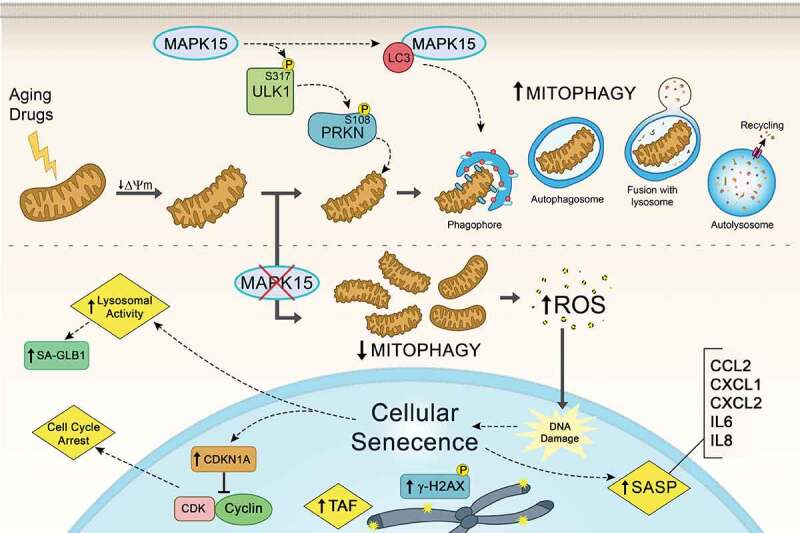
MAPK15 prevents cellular senescence. MAPK15 controls the ability of mammalian cells to efficiently eliminate damaged mitochondria through mitophagy, avoiding the accumulation of dysfunctional organelles that produce increased amounts of reactive oxygen species (ROS), ultimately protecting DNA and other cellular macromolecules from the deleterious effects of oxidative stress. Specifically, MAPK15 positively regulates both basal and toxin-induced mitophagy by stimulating ULK1-dependent PRKN activating Ser108 phosphorylation, contributing to maintain the fitness of the whole mitochondrial compartment. Consequently, reduced levels of MAPK15, in normal as well as in cancer cells, ultimately increase nuclear DNA damage and most markers of cellular senescence, namely cell cycle arrest, CDKN1A/p21 protein levels, histone γ-H2AX and telomere-associated DNA damage foci (TAF) foci, senescence-associated GLB1/β-galactosidase (SA-GLB1/β-Gal) activity and production of different cytokines accompanying the senescence-associated secretory phenotype (SASP). ΔΨm, mitochondrial membrane potential.

Obviously, there are still many questions that need to be addressed about the mechanisms exploited by MAPK15 to control the mitophagic process and the precise chemical and molecular intracellular messengers regulating its activity. Also, in more general terms, it will be certainly interesting to frame MAPK15-dependent stress-responding activities into the pathogenesis of different age-related human diseases. Consequently, this will make it possible to evaluate the potential of using pharmacological and/or genetic modulators of MAPK15 activity to regulate cellular senescence in humans, to increase its potential beneficial effects in cancer or, conversely, reduce its detrimental effects during aging, possibly allowing a better control for different disorders typical of the elderly.
